# Quantitative Reconstruction of Weaning Ages in Archaeological Human Populations Using Bone Collagen Nitrogen Isotope Ratios and Approximate Bayesian Computation

**DOI:** 10.1371/journal.pone.0072327

**Published:** 2013-08-27

**Authors:** Takumi Tsutaya, Minoru Yoneda

**Affiliations:** 1 Department of Integrated Biosciences, Graduate School of Frontier Sciences, The University of Tokyo, Kashiwa, Chiba, Japan; 2 University Museum, The University of Tokyo, Bunkyo, Tokyo, Japan; University of Illinois at Champaign-Urbana, United States of America

## Abstract

**Background:**

Nitrogen isotope analysis of bone collagen has been used to reconstruct the breastfeeding practices of archaeological human populations. However, weaning ages have been estimated subjectively because of a lack of both information on subadult bone collagen turnover rates and appropriate analytical models.

**Methodology:**

Temporal changes in human subadult bone collagen turnover rates were estimated from data on tissue-level bone metabolism reported in previous studies. A model for reconstructing precise weaning ages was then developed using a framework of approximate Bayesian computation and incorporating the estimated turnover rates. The model is presented as a new open source R package, WARN (Weaning Age Reconstruction with Nitrogen isotope analysis), which computes the age at the start and end of weaning, ^15^N-enrichment through maternal to infant tissue, and 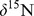 value of collagen synthesized entirely from weaning foods with their posterior probabilities. The model was applied to 39 previously reported Holocene skeletal populations from around the world, and the results were compared with weaning ages observed in ethnographic studies.

**Conclusions:**

There were no significant differences in the age at the end of weaning between the archaeological (2.80±1.32 years) and ethnographic populations. By comparing archaeological populations, it appears that weaning ages did not differ with the type of subsistence practiced (i.e., hunting–gathering or not). Most of 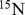-enrichment (2.44±0.90‰) was consistent with biologically valid values. The nitrogen isotope ratios of subadults after the weaning process were lower than those of adults in most of the archaeological populations (−0.48±0.61‰), and this depletion was greater in non-hunter–gatherer populations. Our results suggest that the breastfeeding period in humans had already been shortened by the early Holocene compared with those in extant great apes.

## Introduction

Investigating variations in the breastfeeding and weaning practices of ancient human populations can provide information on the health, cultural traits, and reproduction of these populations. Breast milk provides various antibodies as well as nutrition to infants, and is important for subadult survival [1], [2]. Breastfeeding practices are closely related to the growth of subadults and overall health of a population [3]–[5]. The type of subsistence activities, social constructs, diet and various cultural factors affect breastfeeding practices [6]–[8], and the length of the breastfeeding period is one of the most important determinants of the fertility of a population [9], [10]. Shorter breastfeeding periods tend to result in shorter birth intervals, and, in turn, higher fertility because breastfeeding can delay the resumption of ovulation [11]–[14]. Furthermore, it is supposed that humans are weaned earlier than the other great apes, and understanding evolutionary changes in weaning practices is of great interest [15]–[20].

Stable isotope analysis of bone collagen is useful for reconstructing the dietary habits of ancient people, and it has also been used to reconstruct breastfeeding and weaning practices of archaeological populations (Table S1; [21]–[25]). Nitrogen isotope ratios (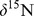 values) of body proteins primarily reflect dietary protein isotope ratios [26], [27]. Prior to and immediately after birth, 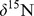 values of infants are the same as those of their mothers [28]. After birth, infants who are exclusively breastfed show 2–3‰ higher 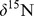 values than their mothers [22], [29] because of the trophic level effect [30]–[32]. Subadult 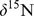 values decrease after the introduction of supplementary foods, and gradually approach the values found in adult bone collagen. It is possible to reconstruct infant feeding practices of an archaeological populations by combining 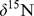 values and physically estimated ages at death of subadults of different ages [5], [33].

However, in previous isotopic studies, weaning ages have been subjectively estimated from visual assessments of detectable changes in subadult bone collagen 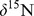 values. To overcome these difficulties, attempts have been made to simulate changes in 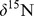 values of subadult bone collagen in two pioneering studies. Schurr [34] used exponential functions to describe changes in 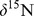 values and estimate the age at the start of weaning. Millard [35] suggested that the model proposed by Schurr [34] suffered from a number of difficulties, and proposed an alternative model that further included a nitrogen mass balance and the age at the end of weaning. However, both models still suffer from the following three problems.

1. The subadult bone collagen turnover rates are not fully considered. The bone collagen turnover rate is high in early infancy [36], [37], but it decreases over the course of subadult growth [38], [39]. If not corrected, the lower bone collagen turnover rates at higher ages would generate significant discrepancies between the visible changes in bone 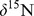 values and actual weaning ages.
2. Some parameters used to describe changes in 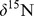 values are determined arbitrarily. Two parameters, 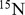-enrichment from maternal to infant tissues and the 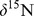 values in weaning foods, could vary among different individuals and populations; therfore, they should be considered as variables in addition to the weaning ages. First, it has been reported that 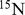-enrichment varies to some extent in modern infant-mother pairs (between 1.7‰ and 2.8‰, 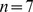: [29]) and in archaeological populations (between 0.5‰ and 4.4‰, 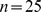: [40]). Second, it is possible that 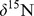 values of materials used in weaning foods were different than those used in adult foods [41], [42].
3. The results are represented as point estimates without either probabilities or confidence intervals. The probabilities of the weaning parameters should be calculated to evaluate the validity of the computation results.

The objective of this study is to develop a model for analyzing cross-sectional 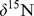 data of subadult bone collagen, and to compare weaning ages between modern ethnographic and archaeological skeletal populations. The model is programmed in R language, which is a free software environment for statistical computing and graphics [43]. The model has the following three important features that are not present in the previous models:

1. The subadult bone collagen turnover rate is estimated anew and incorporated in the equations.
2. The enrichment factor and 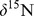 values of weaning foods are included as target parameters to be estimated.
3. Using a framework of approximate Bayesian computation (ABC) allows researchers to calculate the probabilities and credible intervals of the weaning parameters.

### Subadult Bone Collagen Turnover Rate

**Figure 1:**
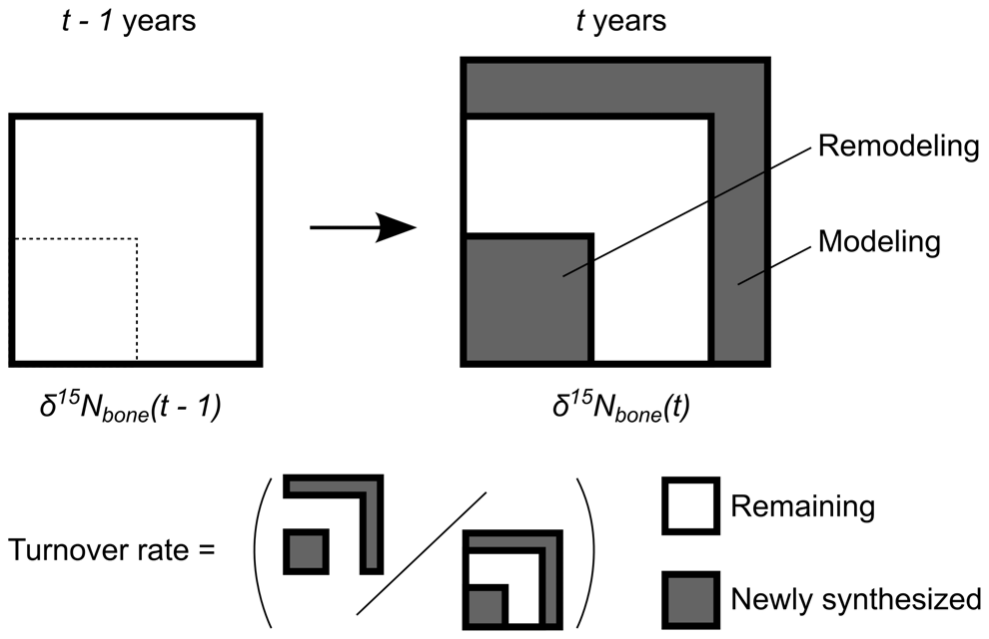
Schematic illustration of the bone turnover process. The 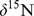 value for bone collagen at the unit time age of 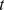 years is represented as 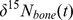.

Temporal changes in the bone collagen turnover rate must be considered to estimate a precise weaning ages from an observed isotope ratio. Bone collagen is laid down during childhood because of bone modeling, which is a formative process primarily associated with skeletal growth, and is replaced throughout life by bone remodeling, which is a coupled resorptive and formative process that does not change the quantity of bone [44], [45]. As indicated in Figure 1, turnover refers to the proportion of newly synthesized bone collagen to the total bone collagen during modeling and remodeling over a unit of time. When the turnover rate is high enough (i.e., ≥1.0 per unit time), bone collagen at a specific age consist only of newly synthesized collagen, and the isotope ratio will immediately change with dietary changes. When the turnover rate is lower (i.e., <1.0), the bone collagen consists not only of newly synthesized but also previously synthesized collagen, the isotope ratio reflects recent and past dietary intakes.

Although temporal changes in turnover rates of subadult bone minerals and collagen have been estimated by analyzing the uptake of 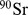 fallout [37], [46] and bomb-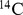 [38], respectively, the estimates produced from these bulk cross-sectional studies were not necessarily precise. Some of the estimates were not based on direct measurements in subadults but on extrapolations from results for adults. In addition, the assumptions made about the dietary intake of tracers in these subadults were simplistic and ignore individual variation. In the present study, we calculated turnover rates from bone metabolism mechanisms at the tissue level so that more precise subadult bone turnover rates could be estimated.

Turnover rates of mineral and organic phases should differ because the mineralization process is much slower than the synthesis of the organic matrix. Bone is a composite material, and is mainly made of a calcified organic matrix [45], [47]. The microstructure of bone material consists of assembled collagen fibrils forming the organic phase, and tiny mineral particles reinforcing them [44]. Two coupled processes are responsible for bone remodeling. The resorptive process involves osteoclasts dissolving the mineral phase by creating a low pH environment around the bone surface, and then producing a lysosomal protease to degrade the organic matrix [48]. The next formative process inolves osteoblasts replacing the organic matrix and rapidly mineralizing it to up to 70% of full mineralization capacity within a few days (primary mineralization), the residual 30% of the mineralization occurring gradually over several years (secondary mineralization) [44], [49]. The mineralization process has been formulated as a mineralization law [49]. Bone modeling occurs with a similar formative process as bone remodeling but with the resorption of the bone cartilage template instead of mineralized old bone [33]. Since the growth [50] and replacement [51] (i.e., turnover, consisting of the modeling and remodeling processes) of bone minerals at the tissue level have been well documented, the turnover of bone collagen can be estimated by correcting the mineralization delay [49].

### Approximate Bayesian Computation

ABC is a modern approach in Bayesian inference that allows posterior distributions to be evaluated when it is difficult to calculate the likelihood function, which describes probabilities under given parameters. Various ABC methods have been applied in diverse fields such as population genetics, evolutionary biology, ecology, and epidemiology [52]–[54].

A general ABC algorithm takes a given observation 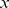 and repeat the following three steps until 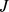 points have been accepted:

1. Draw the candidate parameter 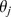 from the prior distribution 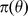.
2. Simulate dataset 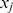 using 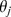 and the model.
3. Accept 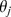 if 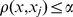, and otherwise reject 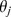.

Here 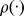 is a function measuring the distance between simulated and observed data points, 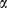 is a fixed tolerance for the “closeness” of simulated and observed data, and 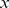, 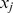, and 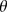 may be vector values. If 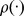 measures appropriate distances and tolerance is sufficiently small, the accepted parameters reasonably approximate the posterior distributions. This is a rejection sampling algorithm, which is the simplest ABC procedure.

Although ABC has proved to be a flexible and powerful approach for evaluating posterior distributions, its major drawback is its inefficiency. Acceptance rates in the simple rejection sampling described above can be very low, especially when the posterior is a long way from the prior, which wastes computing time. Several algorithms have been proposed to increase the sampling efficiency, by introducing weighting with regression analysis [55], [56], Markov chain Monte Carlo sampling [57], and sequential Monte Carlo (SMC) sampling [58]–[60]. We used SMC sampling with corrected partial rejection control proposed by Sisson et al. [59] because this method could be implemented more quickly and simply in our model in the R software environment. SMC sampling is characterized by successively decreasing the tolerance, and weighted resampling from the previous parameter population.

## Results

### Subadult Bone Collagen Turnover Rate

**Figure 2:**
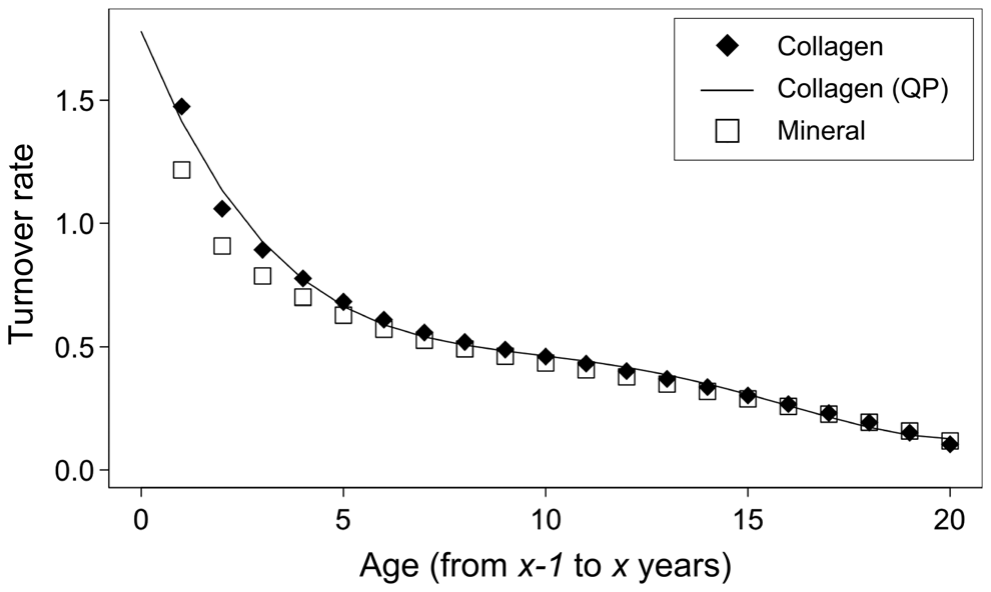
Estimated temporal changes in bone mineral and collagen turnover rates. Turnover rates of bone minerals and collagen are represented as discrete values, and that of collagen is fitted to QP plotted against age.

The calculated turnover rates are shown in Table 1 and Figure 2. The turnover rate of bone collagen was estimated to be larger than that of bone mineral until an individual reaches their late teens, and to decrease over the course of subadult growth. The integrated bone collagen turnover rate from 0.0 to 1.0 years of age was estimated to be 1.588, and the estimated bone collagen turnover rate was higher than 1.000 per year by two years of age (see Table 2). The integrated turnover rate from 0.0 years of age reached 0.966 at 0.60 years of age, suggesting that it takes 31 weeks for infants to fully reflect post-birth dietary 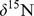 signals. The integrated turnover rate from the age of 19.0 to 20.0 years of age was estimated to be 0.130.

**Table 1:** Estimated temporal changes in turnover rates for bone minerals and collagen.

Age		Turnover rate		
From	To	Mineral	Collagen	Collagen (QP)
0	1	1.217	1.474	1.413
1	2	0.908	1.059	1.134
2	3	0.786	0.892	0.924
3	4	0.700	0.776	0.771
4	5	0.629	0.682	0.664
5	6	0.571	0.611	0.590
6	7	0.527	0.558	0.540
7	8	0.492	0.520	0.507
8	9	0.462	0.489	0.483
9	10	0.434	0.461	0.463
10	11	0.407	0.432	0.441
11	12	0.378	0.402	0.416
12	13	0.349	0.370	0.386
13	14	0.319	0.337	0.349
14	15	0.289	0.302	0.306
15	16	0.258	0.267	0.260
16	17	0.227	0.231	0.213
17	18	0.194	0.193	0.171
18	19	0.158	0.151	0.139
19	20	0.118	0.104	0.124

QP: calculated from the QP function.

**Table 2:** Summary of MDEs for the archaeological populations calculated using the developed model.

Parameter	Mean	SD	Median	n	Weighted mean
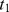 (year)	1.07	0.78	0.95	34	0.94
*t*_2_	2.80	1.32	2.55	34	2.10
*E* (‰)	2.44	0.90	2.50	37	2.30
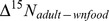 (‰)	−0.48	0.61	−0.55	38	−0.41

Weighted means are calculated from all 38 MDEs, including those with probabilities less than 0.0025 (joint probability) or 0.05 (marginal probability).

### The Implemented Model

The model developed in the present study is distributed as the R package WARN (Weaning Age Reconstruction with Nitrogen isotope analysis: [61]), which is freely available under the GPL license and can be downloaded from the Comprehensive R Archive Network (see also [43]). Although they are not considered in the present study, credible intervals can be calculated for a given parameter range using the WARN package. Images of the results calculated using the package are shown in Figure S1.

### Application of the Model

The maximum density estimators (MDEs, i.e., weaning parameters that result in maximum probability density), posterior probabilities, and the other information from each archaeological dataset are given in Table S1. To standardize the results, the estimated 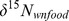, which is the 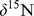 value of collagen synthesized entirely from weaning foods, is represented as a difference from the mean 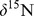 value for total adults, 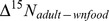.

**Figure 3:**
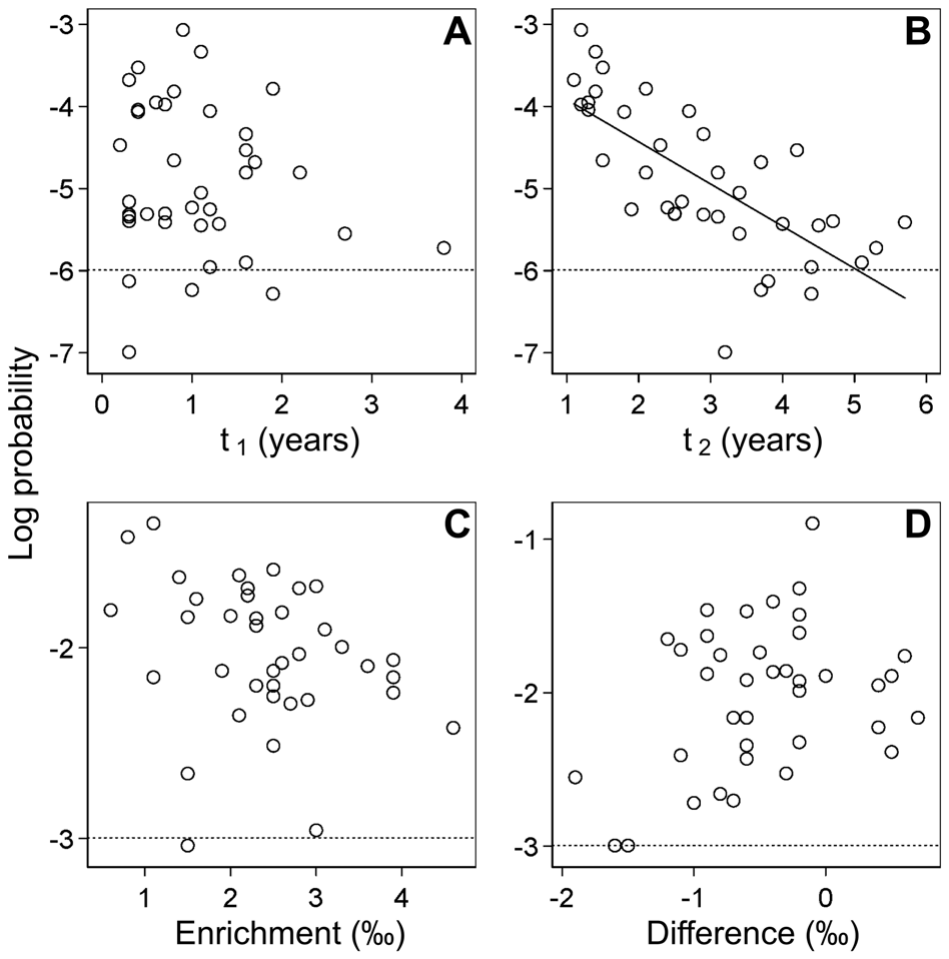
Relationships between MDEs and the logarithms of the probabilities of the four weaning parameters for the archaeological populations. Dotted lines indicate the thresholds of probability. (A) and (B) The age at the start and end of weaning, respectively. (C) 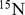-enrichment from maternal to infant tissue. (D) The difference between the 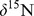 value of collagen synthesized entirely from weaning foods and the mean 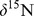 value for all adults. A regression line is also shown for 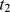 (slope = −0.514, intercept = −3.403).

Results for the Bjärby population [62] were excluded because 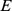 for the population was estimated to be negative (i.e., −1.6‰, see Table S1). There was only one individual less than three years of age in the Bjärby population, so this biologically unexpected temporal change pattern in the 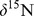 value appears to be an artifact. Relationships between MDEs and the logarithmic probabilities are shown in Figure 3. A negative linear correlation was found between the age at the end of weaning, 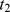, and the logarithm of the joint probability of the weaning ages (Spearman’s rank correlation test: 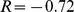, 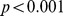) but the other parameters did not indicate evident relationship. Assuming that MDEs with probabilities of less than 0.05 (0.0025 for the joint probability of the weaning ages) were unlikely, they were also excluded from the subsequent analyses. A Kruskal–Wallis test indicated that the MDE sets did not differ by the type of bone analyzed (rib, a combination of a rib and another, and those other than ribs; 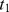: 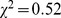, 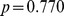; 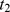: 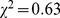, 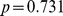; 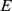: 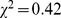, 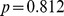; 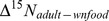: 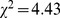, 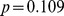). Although the parameters have credible intervals (see Figure S1 for an example), only MDEs are considered in the subsequent analyses to simplify the discussion.

**Figure 4:**
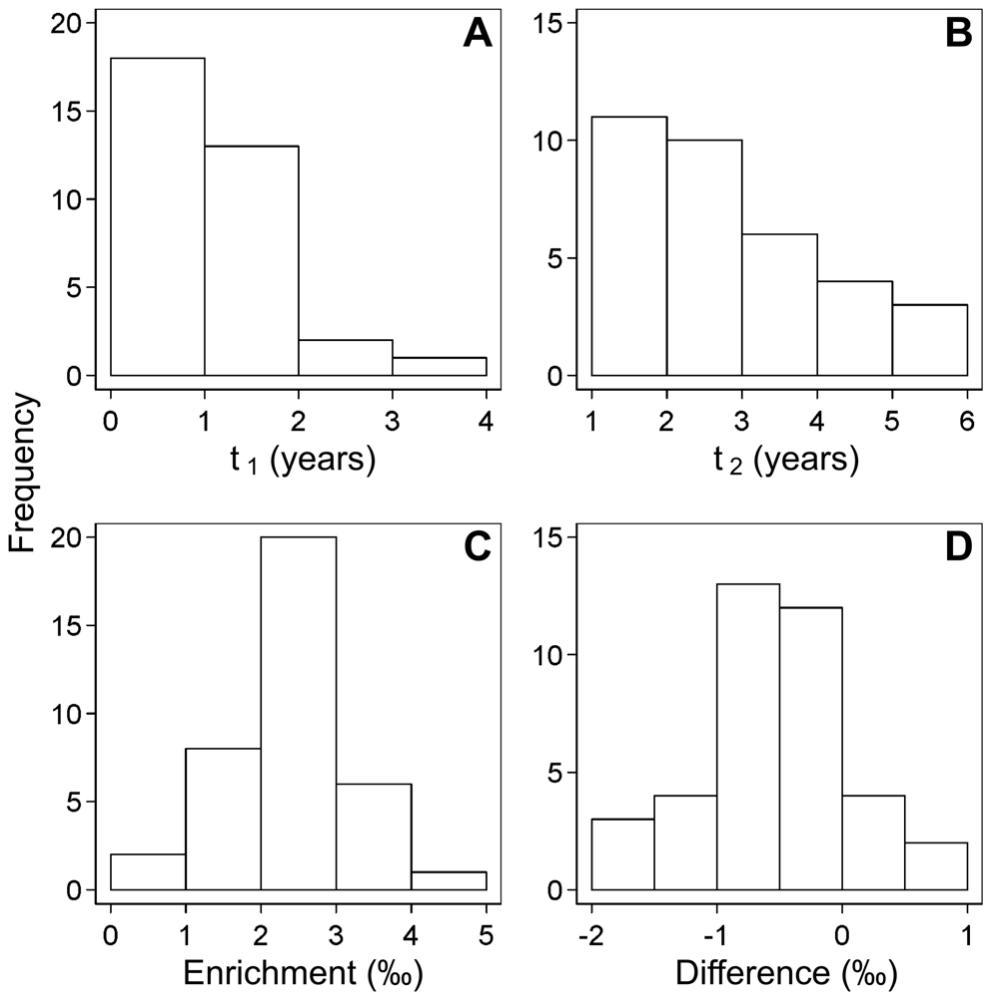
Histograms for the MDE distributions of the four weaning parameters for the archaeological populations. See Figure 3 for the meanings of the labels.

Histograms of the MDE parameters are shown in Figure 4, and summarized in Table 2. In MDEs of 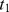 and 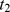, 18 (52.9%) and 21 populations (68.8%) is in the range between 0.0 and 1.0 years and between 1.0 and 3.0 years of age, respectively, and frequencies decrease with age. MDEs of the other two parameters distribute on the both sides of the mode, and bands ranging between 2.0 and 3.0‰ and between −1.0 and −0.5‰ represented modes for 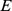 and 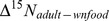, respectively. The means of all of the 38 parameters weighted by their probabilities, including parameters with probabilities below 0.0025 (joint probability of the weaning ages) or 0.05, are also shown in Table 2.

**Figure 5:**
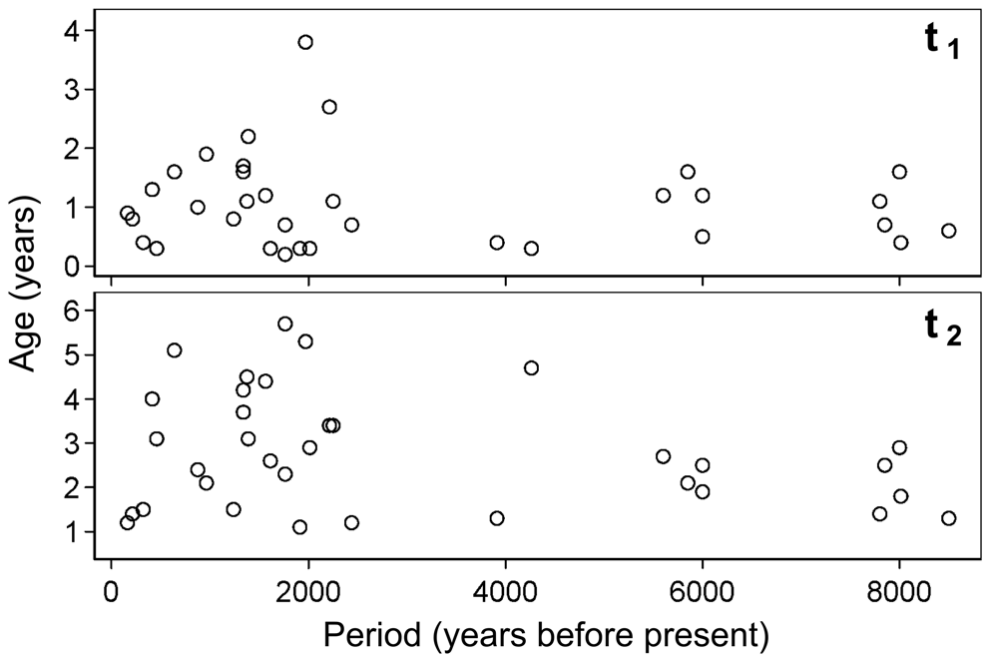
Maximum density weaning ages plotted by the midpoint time period for the populations.

The estimated weaning ages in various archaeological populations were compared in terms of the time period when the population were alive and the type of subsistence. No relationship was found between weaning ages and the midpoint time when the populations were alive (Figure 5). Mann–Whitney U-tests did not indicate significant differences in 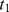, 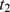, 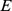, and 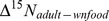 between hunter–gatherer (HG) and non-hunter–gatherer (NHG) populations (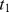: 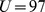, 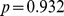; 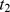: 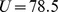, 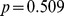; 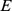: 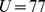, 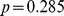; 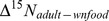: 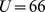, 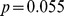), but did indicate a large difference for 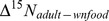. The mean and SD for 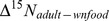 values of the HG and NHG populations were −0.14 ±0.40‰ and −0.57±0.63‰, respectively. The mean and standard deviations (SDs) of MDEs by type of subsistence are shown in Table 3.

**Table 3:** Mean and SDs of MDEs for the archaeological populations by the type of subsistence.

Subsistence	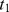 (year)	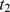 (year)	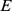 (‰)	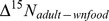 (‰)
Hunting–gathering	0.93±0.37	3.01±1.25	2.54±0.68	−0.14±0.40
Non-hunting–gathering	1.11±0.85	2.74±1.35	2.38±0.94	−0.57±0.63

**Figure 6:**
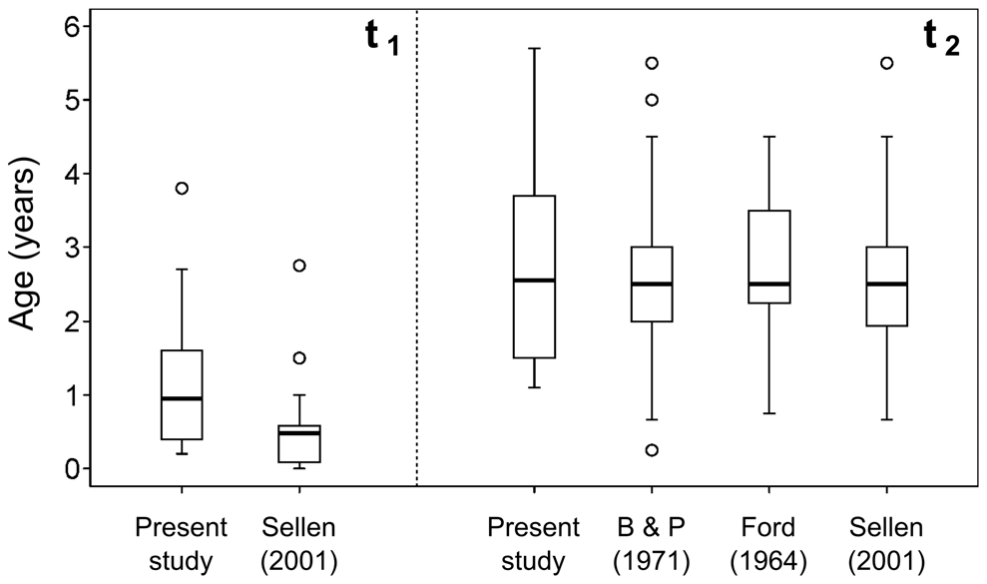
Boxplots for the weaning ages estimated in this study and observed in previous ethnographic studies. Data were obtained from Barry and Paxson (1971): [88], Ford (1964): [7], and Sellen (2001): [89].

MDEs for the weaning ages of the archaeological populations were compared to those obtained from ethnographic studies (Table 4) as illustrated in Figure 6. A Mann–Whitney U-test indicated that the ethnographic populations were significantly younger (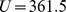, 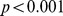) at the start of weaning (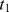). However, the Kruskal–Wallis test showed no significant differences in the age at the end of weaning among archaeological and ethnographic populations (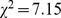, 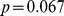).

**Table 4:** Summary of ages at the start and end of weaning found in previous studies of ethnographic populations.

Weaning age (year)	Mean	SD	Median	n	Reference
Start	0.48	0.50	0.48	42	[89]
End	2.63	0.98	2.50	156	[88]
	2.72	0.72	2.50	44	[7]
	2.39	0.87	2.50	108	[89]

## Discussion

### Bone Collagen Turnover Rate

Tracer intake and biochemical marker studies have shown that the bone mineral and collagen turnover rates are high in the first few years of life (i.e., >1.0 per year) [36], [37], [39], which is consistent with our results (see Table 1 and Figure 2). However, temporal changes in the bone collagen turnover rate after infancy and before adulthood have never been estimated directly and continuously, and the present study allowed them to be estimated. An isotopic study on an archaeological infant of a known age has suggested that infant rib bone collagen can fully reflect post-birth dietary 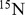 input, in an extreme case, in only five to six weeks [63], but this is estimated to take 31 weeks from our results. Our study allows typical temporal changes to be estimated, but the bone collagen turnover rate in subadulthood probably varies.

The integrated bone collagen turnover rate from 19.0 to 20.0 years of age was estimated to be 0.130 per year in our study (see Figure 2), which is a little higher than that proposed by Stenhouse and Baxter (10.4±2.7% during adulthood, [64]) and Hedges et al. (9.7% and 4.1% for 20-year-old male and female femora, respectively, [38]). Although the type of bone sampled by Stenhouse and Baxter [64] is not stated, differences between the turnover rates in different bone types could cause these different results. The turnover rates are higher in bones with greater surface to volume ratios than those in bones with smaller ratios [65]. Ribs, which were target bones in our study, have relatively high proportions of cancellous and thin cortical parts, whereas femur analyzed by Hedges et al. [38] has a lower proportion of cancellous and thick cortical parts. although there are slight differences, the overall trend of the temporal changes in bone turnover rates in this study is consistent with previous estimates.

### Validity of the Model

Although it is desirable to test the model validity, the absence of proper test data means this is not possible. Archaeological skeletal populations cannot be tested because the true weaning ages are usually unknowable, and historical literature, if any, describing breastfeeding practices at the time period when the population lived sometimes differs from actual practices (e.g. [63], [66]; see also [41], [67], [68]). Since the model presented here was intended for human subadult bones, conducting an experimental study was difficult, and hair, nail, and other tissues were not suitable for analysis because they have different turnover rates than bone collagen. Experimental studies of animals other than human would not be appropriate because human growth patterns are unique among mammals [18], [69]; therefore the nitrogen mass balance in human subadults would probably be different from that in other animals.

Although there is no way of testing the validity of the model under existing conditions, the present model provides advantages in the objective comparison of weaning parameters among different populations. For example, lower and higher 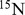-enrichment values of infants than the biologically expected values of 2 to 3‰ [29] have been reported in Wetwang [70] and Isola Sacra [68] populations, and these results are objectively shown to be 0.8‰ and 4.6‰, respectively, by the model presented here (see Table S1).

### Weaning Practices in Archaeological Populations

Assuming that lower probabilities for the weaning parameters suggests greater individual variability, a negative linear relationship between the MDEs of 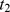 and the logarithms of their probabilities can be interpreted from two perspectives. First, early end-of-weaning departures from the norm would increase with the norm of older weaning age; therefore, individual variations within the population would increase. Second, the subadult bone collagen turnover rate decreases as their age increases (see Figure 2) and the bone takes longer to reflect isotopic changes in dietary input, which would result in individual variations in the weaning age being amplified. Another interpretations could be made on MDEs of 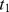 that they are so small that the two factors just mentioned have little effect on the relationship between MDEs of 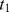 and its probabilities.

Some bioarchaeological studies have led to the hypothesis that breastfeeding duration become shorter around the time of the subsistence transition from hunting–gathering to agriculture during the Holocene mainly because of the availability of weaning food [71]–[73], but no significant differences were observed between HG and NHG population weaning ages. Furthermore, there were no consistent trends of the secular changes in the estimated MDEs of weaning ages (Figure 5). These results suggest that the type of subsistence and time period are not determining factors for weaning ages in a population. The hypothesis of shortened breastfeeding periods in NHG populations is not supported by an ethnographic meta analysis [74], and the results reconstructed from archaeological populations in the present study support this conclusion. However, 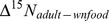 values were greater in NHG populations than those in HG populations although the difference is not significant. Assuming that the cause of this difference is dietary, this could be interpreted as indicating that NHG people fed their subadults more lower trophic level weaning foods, such as plants, than HG people did. Although the type of subsistence did not determine the weaning ages, it could have affected the weaning food used.

MDEs of 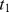 were significantly higher than those reported for ethnographic populations (Figure 6), and also higher than that required biologically for infant health (i.e., beginning at the age of six months, [2]). This difference would have been caused by a discrepancies in the intended “age at start of weaning” among ethnographic, nutritional, and isotopic studies rather than a reflection of actual weaning practices in the past. The 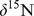 values in bone collagen mainly reflect the ratios of dietary protein [26], [27], but nutritionists and cultural anthropologists see the age when liquid or solid foods are first introduced as the start of weaning, without considering the protein contribution from these foods [3], [74]. Subadult nutritional requirements are not necessarily met by protein, and high-protein foods are often avoided as supplementary foods, especially in early part of the weaning process [75], [76]. It would be valid to assume that the actual age when solid or liquid foods were introduced could be lower, but that the subadult bone collagen 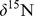 value did not record the event.

There were no significant differences in the age at the end of weaning between archaeological and ethnographic populations, and our results suggest that the age at the end of weaning in human populations without modern artificial baby foods did not change in a consistent manner throughout the Holocene. However, although results from observational and isotopic studies are not necessarily directly comparable, the estimated mean MDE of 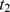 (2.80±1.32 years) is still smaller than the age at the end of weaning in great apes (3.0 years for gorillas, 4.8 years for chimpanzees, and 6.0 years for orangutans, [16]). Shortening of the breastfeeding period would have occurred during the process of human evolution in the Pleistocene. Existing nitrogen isotope analysis for reconstructing weaning practices requires many well-preserved subadult bones, new analytical methods, such as stable isotope analysis of serial sections of tooth dentin [77], [78], and enamel [79] and the analysis of Sr/Ca ratios in tooth enamel [80], could allow weaning ages to be reconstructed for more ancient hominins.

Although it is possible that the prior distribution affect the result, set of MDEs of 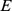 (mean: 2.44±0.90‰) is consistent with biologically valid values. Isotope analysis on modern human mother and infant pair have reported that the 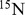-enrichment is in the range between 1.7 and 2.8‰ [29]. Reconstructed 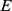 vary to some extent (Figure 4C), and this would possibly be stem from variations in mother’s diet [68], [81] and/or physiological factor affecting nitrogen mass balance such as growth [40], nutritional adjustment in metabolism [82], pathology [83], and drought [84].

Most of the 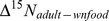 values were negative, which could be interpreted as having dietary or physiological causes. First, it is probable that the proportion of foods from lower trophic levels with lower 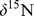 values was universally greater in the subadult than that in the adult diet. It has been reported in ethnographic studies that lower trophic-level foods such as cereals and legumes are preferred to higher trophic-level foods such as animal and fish foods as weaning foods in various populations [7], [74]–[76], [85], and animal milk would not be universally available as a weaning food in the ancient period. Second, a positive nitrogen balance in developing subadults would probably be one of the reasons for lowered tissue 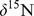 values [28]. Although the effect of growth is not evident in the long bones of juvenile and adolescents from seven to nineteen years of age [40], we studied rib bones of younger subadults. The turnover rate in younger subadult bones would be greater than that in juvenile and adolescent bones (see Figure 2); therefore, it is possible that the effect of growth on the 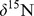 values is recorded and evident in the present study.

Finally, there are two caveats to consider before applying the model presented here. First, the present model is intended for bones with relatively high turnover rates, such as cancellous bones or ribs. Although WARN was applied equally to isotopic data from bones with relatively low surface to volume ratios (e.g., limbs, cranium, and mandible) to maximize the sample size in the present study, attention to this aspect is required for more precise analysis. Second, the WARN approach will always attempt to fit a model, even if the subadult 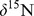 values do not indicate breastfeeding and weaning signals. If researchers cannot find patterns of isotopic changes by visually inspecting the data, they are urged to examine their data carefully before applying the model, for example, for a biased age distribution or high isotopic variability in subadults. Although the estimated turnover rate and model developed can be further improved, in this study, we propose a framework for objectively and quantitatively analyzing and interpreting subadult bone collagen 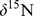 values. A precise reconstruction of past breastfeeding and weaning practices over a wide range of time periods and geographic regions could make it possible to understand this unique feature of human life history and cultural diversity in infant feeding practices [15]–[20].

## Materials and Methods

### Terminology

**Figure 7:**
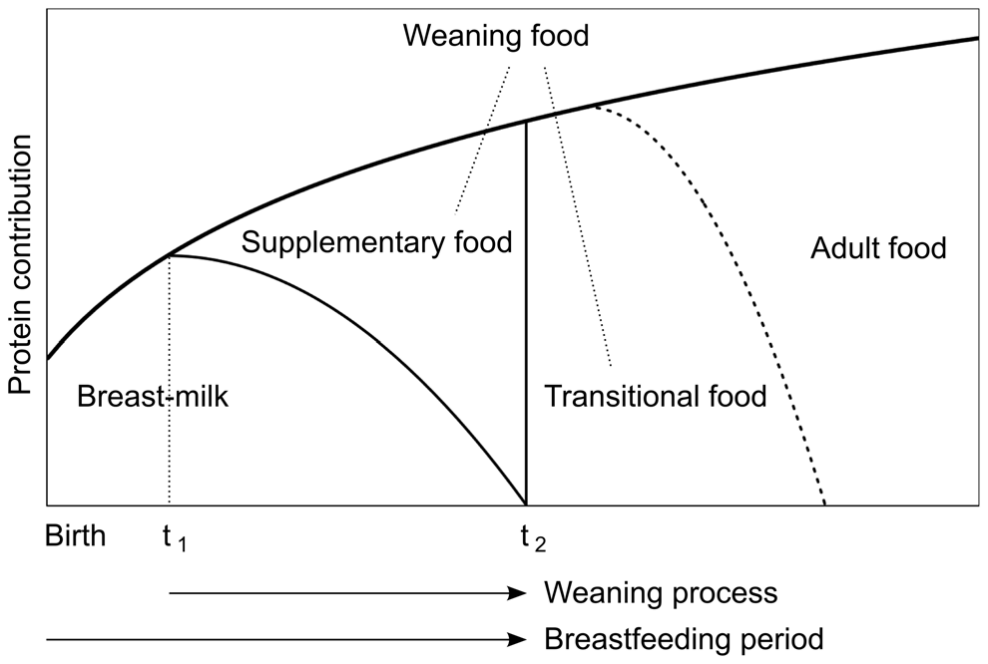
Schematic illustration of the terminology used for the subadult diet. The ages at the start and end of weaning are represented as 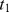 and 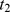, respectively.

A lack of consistency in the use of terminology limits the usefulness of weaning studies and makes it difficult to compare studies; therefore, we defined the terms on the basis of definitions proposed by Dettwyler [3] and WHO [86]. “Weaning” is defined as a process consisting of a starting age (the first time that food other than mother’s milk is constantly included in the diet) and an ending age (the time when mother’s milk stops being constantly consumed). “Supplementary food” means foods other than mother’s milk that is consumed by subadults during the weaning process, and this food can be isotopically different from adult foods. “Adult food” means the typical foods consumed by adults in the population. “Transitional food” means foods that is isotopically different from adult food and is consumed after the weaning process, but it is possible that no transitional foods are consumed by a specific population. “Weaning food” is a generic name of supplementary and transitional foods. These food types are evaluated by their dietary protein contributions from the nitrogen isotope analysis of bone collagen. These definitions are illustrated schematically in Figure 7.

### Estimating Subadult Bone Collagen Turnover Rates

In this study, bone collagen turnover rates in subadults were calculated from the modeling [50] and remodeling [51] rates for cancellous bone minerals, and the mineralization law for the bone organic matrix [49]. “Turnover” is defined as the aggregated effects of bone modeling (i.e., the addition of bone tissue by skeletal growth) and remodeling (i.e., the replacement of existing bone tissues). Here we present a summary of the procedures, and the detailed mathematical expressions are given in section 1 of the Text S1. First, following Leggett et al. [51], the bone mineral turnover rate 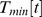 over one unit of time (i.e., one year from 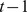 to 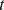, Equation S1–4) in childhood was calculated using the functions that describe the temporal change in bone mineral mass 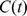 [50] (Equation S1–1) and the remodeling rate 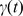 [51] (Equation S1–2). Next, the bone collagen turnover rates 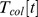 over one unit of time from 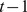 to 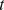 years (Equation S1–5) were calculated sequentially with 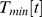 and the mineralization law, 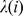, which described the bone collagen mineralization process [49]. The mineralization law was derived from Ruffoni et al. [49], and represents the rate of mineralization of the collagen portion at the 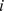
th year after the collagen matrix was formed (Equation S1–3). Finally, the resulting discrete turnover rates were coerced into a quartic polynomial (QP) formula (Equation S1–6). Turnover rates at ages less than one year were extrapolated from the QP function.

### Changes in 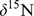 Values of Diet and Bone Collagen

Following Millard [35], the 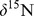 value of newly synthesized collagen at a given age of 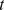 years was defined by four parameters, the ages at the start (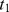) and end (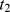) of weaning, enrichment factor between the infant and mother (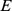), and 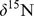 value of collagen synthesized entirely from weaning foods (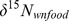) (Equation S2–1 and S2–2). The 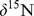 value of newly synthesized collagen equals the sum of the 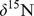 value of the mothers tissue and enrichment factor before weaning (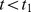), which changes exponentially during weaning (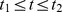), and equals the collagen 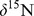 value that fully reflects the consumption of supplementary food (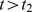).

Then, the incorporation of newly synthesized collagen and replacement of existing collagen in bone are simulated in over each successive unit time using the estimated turnover rate for bones (Equation S1–6 and S2–3). As most isotopic studies on weaning have focused on rib bones, because of their assumed fast turnover [65] and relatively trivial importance in morphological studies (see Table S1), the rate incorporated into the present model was that of cancellous bones. Although the rib bones that were sampled would have contained cortical parts, the relatively high surface to volume ratio in ribs would have resulted in a high proportion of cancellous parts and only thin cortical parts, making the turnover rate comparable to that of cancellous bones [65]. Although one unit of time consists of one year, adjustments from the last unit of time enables simulated 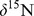 values to be calculated for each individual in the dataset (Equation S2–4 and S2–5). Simulated 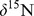 values, 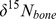, for each individual can be calculated under the given weaning parameters (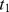, 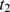, 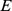 and 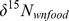) using the model described above. The most appropriate weaning parameters can be estimated by minimizing the mean least square distance between the observed and resultant simulated change in bone collagen 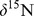 values. The equations used to model the isotopic changes in subadult bone collagen during the weaning process are described in detail in section 2 of the Text S1.

### Incorporation of ABC

To obtain posterior probabilities of the estimated parameters, fitting calculations between the observed and simulated data are performed under the ABC framework with SMC sampling. The details of the SMC procedures proposed by Sisson et al. [59] that were used in our model are given in section 3 of the Text S1. Using the ABC framework, a number of weaning parameter sets that give well-fitted 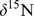 values were sampled and assumed to represent the posterior distributions of the parameters. After applying the ABC procedure, posterior distributions were smoothed using the kernel density estimation [87], and joint probabilities for weaning ages (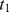 and 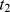) and marginal probabilities for 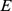 and 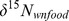 were calculated. In the density estimation, posterior probabilities were calculated to one decimal places for discrete parameter categories because strictly implementing the density estimation as a continuous distribution requires advanced numerical analysis techniques.

### Application to Archaeological Populations

The target populations were chosen according to the following criteria: (1) the numerical values for age and bone collagen 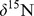 were reported and (2) more than six subadult individuals were included. The mean and SD of adult females and all adults were calculated anew if their individual numerical values were reported, and otherwise the summarized values indicated in article, table, or figure were used. Data from teeth were excluded. The 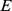 and 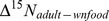 (the difference between the 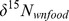 and mean 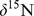 value for all adults) values were estimated on the basis of the mean 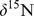 values of adult females and total adults, respectively. If there were no mean 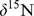 values for adult females, the values for total adults were used instead, and *vice versa*. Information on the time period when the population lived and the type of subsistence practiced (hunting–gathering or not) were extracted from the literature. For the purposes of comparison, weaning ages that had been reported numerically in three independent meta-analyses of the ethnographic literature were used [7], [88], [89]. Although there would been overlap among the target ethnographic populations and problems relating the non-independence of cultures (i.e., the Galton’s problem [90]) for both the archaeological and ethnographic populations, we treated them separately.

The model was applied to 39 archaeological populations that had been reported in 29 different publications (Table S1). Since we targeted diet in early life before adolescence, the age of the individuals used in this study was limited to below 10 years. The prior distributions were set to have normal distributions with means of {0.5, 3.0, 1.9, and 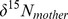} and SDs of {3.0, 3.0, 0.9, and 3.0} for 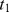, 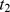, 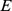, and 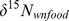, respectively, except for one population. For the Isola Sacra population [68], the prior distributions were set to have means of {0.0 and 1.0} and SDs of {1.0 and 1.0} for 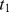 and 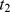, respectively, because this dataset had the unique features of younger weaning ages and higher 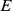 values, which complicated the parameter optimization. The mean weaning age was obtained from the ages recommended by modern pediatricians and the biologically expected ages [91]. The mean and SD values for 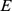 was obtained from values reported by Waters-Rist and Katzenberg [40]. The number of “particles” (i.e., unit sets of weaning parameters resampled) was 10000. The number of parameter “populations” (i.e., units of successively reducing tolerance) was seven.

### Statistics

All statistical calculations were performed using R software version 3.0.0 [43].

## Supporting Information

Figure S1
**An example of the results of applying WARN model using the Spitalfields population as a case study.** (A) Modeled temporal changes in the 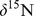 values by subadult age calculated from the reconstructed MDEs. Mean and SD ranges for adult females and all adults are indicated with open circles and crosses, respectively. (B) Contour lines show the posterior probability for the combination of weaning ages. The target ranges for 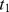 and 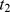 are 0.0–1.2 years and 1.2–2.0 years of age, respectively, and the calculated joint probability for the ranges is 0.942. (C) Distribution of posterior probabilities for the 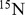-enrichment from maternal to infant tissues. The target range is 1.6–2.4‰, and the calculated marginal probability for the range is 0.961. (D) Distribution of posterior probabilities for the 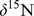 values for collagen synthesized entirely from weaning foods. The target range is 12.4–13.0‰, and the calculated marginal probability for the range is 0.960. Subadult ages and bone collagen 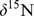 values were obtained from Nitsch et al. [63], [92].(TIFF)

Table S1 **Summary of model application results and descriptions of the archaeological populations intended in present study.**
(PDF)

Text S1 **Detailed procedures and mathematical expressions in the estimation of subadult bone collagen turnover rate and the WARN program.**
(PDF)
