# Correction: Quantitative Reconstruction of Weaning Ages in Archaeological Human Populations Using Bone Collagen Nitrogen Isotope Ratios and Approximate Bayesian Computation

**DOI:** 10.1371/journal.pone.0119778

**Published:** 2015-03-16

**Authors:** The PLOS ONE Staff

There are errors in the equations of file S1 Text of the published article. Please view the corrected file S1 Text here.

## Supporting Information

S1 Text Detailed procedures and mathematical expressions in the estimation of subadult bone collagen turnover rate and the WARN program. (PDF)
