# Supplementary figures and images for: Quantitative Reconstruction of Weaning Ages in Archaeological Human Populations Using Bone Collagen Nitrogen Isotope Ratios and Approximate Bayesian Computation
Source: PLoS One. 2013 Aug 27;8(8):e72327. doi: 10.1371/journal.pone.0072327 (PMC3754991; doi:10.1371/journal.pone.0072327)

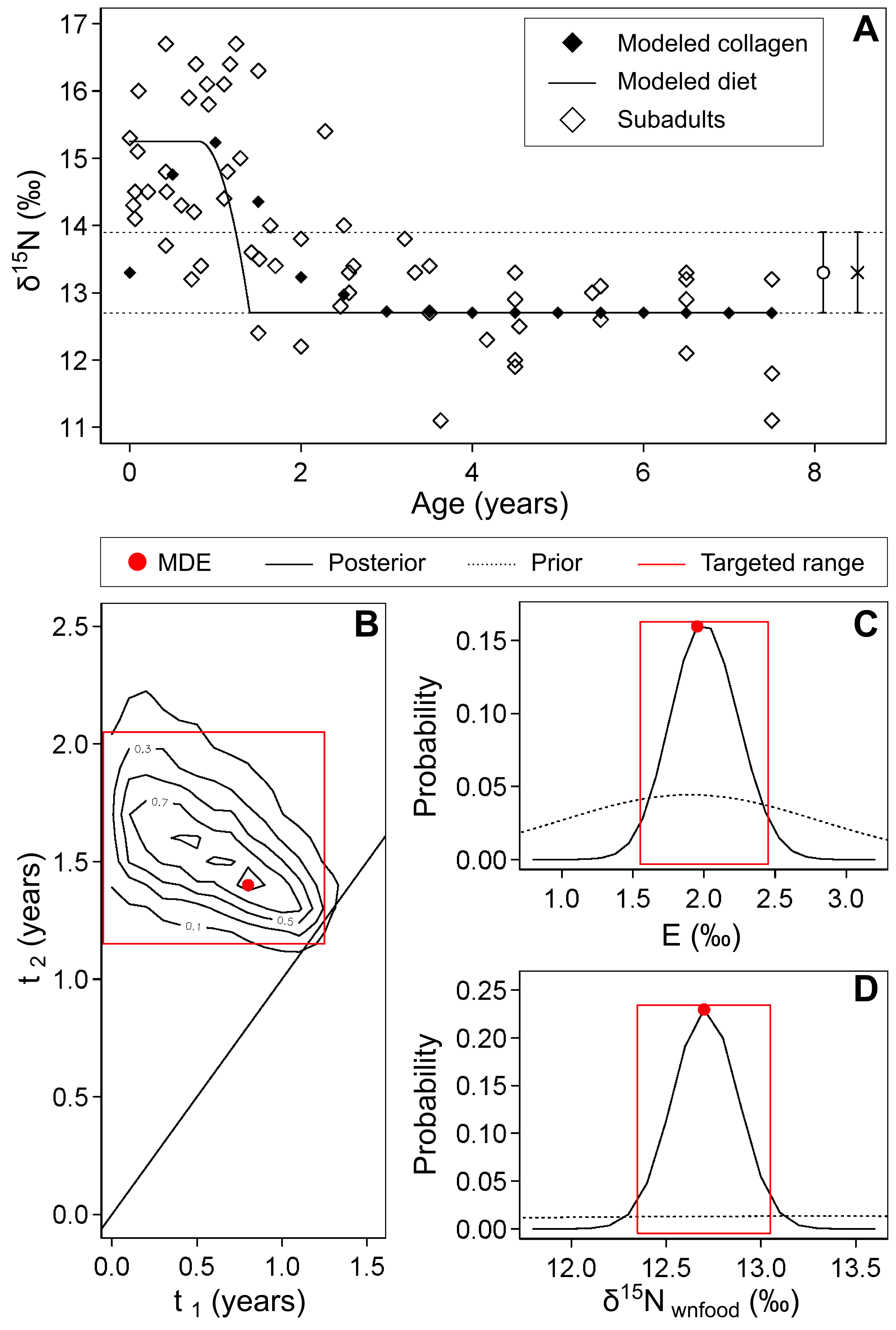

Supplement: Figure S1 — An example of the results of applying WARN model using the Spitalfields population as a case study. (A) Modeled temporal changes in the values by subadult age calculated from the reconstructed MDEs. Mean and SD ranges for adult females and all adults are indicated with open circles and crosses, respectively. (B) Contour lines show the posterior probability for the combination of weaning ages. The target ranges for and are 0.0–1.2 years and 1.2–2.0 years of age, respectively, and the calculated joint probability for the ranges is 0.942. (C) Distribution of posterior probabilities for the -enrichment from maternal to infant tissues. The target range is 1.6–2.4‰, and the calculated marginal probability for the range is 0.961. (D) Distribution of posterior probabilities for the values for collagen synthesized entirely from weaning foods. The target range is 12.4–13.0‰, and the calculated marginal probability for the range is 0.960. Subadult ages and bone collagen values were obtained from Nitsch et al. [63], [92]. (TIFF) [file pone.0072327.s001.tiff]
