# Supplementary material for: Quantitative Reconstruction of Weaning Ages in Archaeological Human Populations Using Bone Collagen Nitrogen Isotope Ratios and Approximate Bayesian Computation
Source: PLoS One. 2013 Aug 27;8(8):e72327. doi: 10.1371/journal.pone.0072327 (PMC3754991; doi:10.1371/journal.pone.0072327)
Supplement: Table S1 — Summary of model application results and descriptions of the archaeological populations intended in present study. (PDF) [file pone.0072327.s002.pdf]

Table S1. Summary of model application results and descriptions of the archaeological populations intended in present study.

ID	Site	Location	Period	Midpoint period (BP)	Bone element	Subsistence
1	Ajdovska jama cave	Slovenia	6400–5300calBP	5850	NA	NHG
2	Angel	Ohio, USA	AD1300–1450	638	Long bone	NHG
3	Charlston Annis	Ohio, USA	3500–1000BC	4263	Long bone	HG
4	Aşıklı Höyük	Turkey	9000–8000calBP	8500	Rib	NHG
5	Baikal	*Cis*-Baikal, Siberia	9000–3000BP	6000	NA	HG
6	Bjärby	Sweden	AD0–200	1913	Cranial, limb	NHG
7	Çatalhöyük	Turkey	7400–8300BP	7850	NA	NHG
8	Çayönü Tepesi	Turkey	9000–7000calBP	8000	Rib	NHG
9	Conchopata	Peru	AD550–1000	1238	Cranial, limb, rib	NHG
10	Dorset late Iron Age	UK	100BC–AD100	2013	Rib	NHG
11	Dorset Romano-British	UK	AD43–	1970	Rib	NHG
12	Fushimi	Japan	AD1600–	413	Rib	NHG
13	Harvie	Ontario, Canada	1825–1892	154.5	Rib	NHG
14	Isora Sacra	Italy	AD0–200	1913	Rib	NHG
15	Kastella	Greece	AD1000–1100	963	Rib	NHG
16	Kellis	Egypt	AD250	1763	Limb, rib	NHG
17	Indian Knoll	Ohio, USA	3500–1000BC	2250	Long bone	HG
18	Kulubnarti R	Sudan	AD550–800	1338	Rib	NHG
19	Kulubnarti S	Sudan	AD550–800	1338	Rib	NHG
20	Leptiminus	Tunisia	AD100–400	1763	Long bones, rib	NHG
21	Lerna	Greece	2100–1700BC	3913	Rib	NHG
22	Lokomotiv	*Cis*-Baikal, Siberia	8800–7000/6800calBP	7800	Limb	HG
23	Matjes River Rcok Shelter	South Africa	12000BP–recent	6000	Cranial, limb, rib	HG
24	McPherson	Ontario, Canada	AD1530–1580	458	Rib	NHG
25	Meuse Basin	Belgium	10000–2000BC	8013	Clavicle, jaw, long bones	NHG
26	Marco Gonzalez and San Pedro	Beliz	100BC–AD1350	1388	Rib	NHG
27	Newark Bay	UK	550–1200BP	875	Rib	NHG
28	Nukdo	South Korea	550–300BC	2438	Cranial, limb, rib	NHG
29	Prospect Hill	Ontario, Canada	1824–1879	161.5	Rib	NHG
30	Queenford Farm	UK	AD300–500	1613	Limb, rib	NHG
31	Shamanka II	*Cis*-Baikal, Siberia	8800–7000/6800calBP	7800	Limb	HG
32	Spitalfields	UK	AD1700–1900	213	Rib	NHG
33	Sully	South Dakota, USA	1650–1733	321.5	Rib	NHG
34	Tinslay Hill	Ohio, USA	AD1300–1450	638	Long bone	NHG
35	Triberga	Sweden	AD500–1000	1263	Limb	NHG
36	Ust'-Ida I	*Cis*-Baikal, Siberia	6000/5800–5200calBP	5600	Limb	HG
37	Wetwang	UK	300-100BC	2213	Cranial, flat bone, rib	NHG
38	Yeanri	South Korea	AD300-600	1563	Cranial, limb, rib	NHG
39	Yukisma	California, USA	2200-250BP	1375	Rib	HG

ID	n	MDE					Probability			Adult female		Total adult		Reference
		t_1	t_2	E	$\delta^{15}N_{wnfood}$	$\Delta^{15}N_{adult-wnfood}$	t_1 and t_2	E	$\delta^{15}N_{wnfood}$	Mean	SD	Mean	SD	
1	12	1.6	2.1	2.5	7.5	0.0	0.00818	0.111	0.151	8.3	0.69	7.5	1.43	[1]
2	16	1.0	3.7	2.3	7.7	-0.6	0.00196	0.111	0.115	8	0.8	8.3	0.7	[2]
3	24	0.3	4.7	2.5	6.7	-0.3	0.00453	0.204	0.080	6.7	1.4	7	1.3	[2]
4	13	0.6	1.3	2.9	10.0	0.4	0.01923	0.103	0.108	9.6	0.91	NA	NA	[3]
5	21	0.5	2.5	1.5	12.3	-0.2	0.00494	0.048	0.137	13.3	2.37	12.5	1.98	[4]
6	8	2.1	3.3	-1.6	13.9	0.5	0.00566	0.130	0.184	13.2	0.4	13.4	1.4	[5]
7	28	0.7	2.5	1.1	10.1	-0.9	0.00497	0.116	0.153	10.8	0.92	11	0.92	[6]
8	17	1.6	2.9	3.9	5.4	-0.6	0.01307	0.116	0.096	6	0.53	NA	NA	[3]
9	8	0.8	1.5	3.9	10.2	-0.4	0.00949	0.127	0.155	10.6	0.99	10.6	1.15	[7]
10	6	0.3	2.9	1.5	7.9	-1.5	0.00491	0.070	0.050	9.4	0.51	9.4	0.61	[8, 9]
11	8	3.8	5.3	0.6	8.6	-0.8	0.00327	0.165	0.070	9.5	1.16	9.4	0.92	[8, 9]
12	6	1.3	4.0	1.1	11.5	-0.5	0.00438	0.260	0.176	11.9	0.69	12	0.6	[10]
13	6	1.9	4.4	1.4	10.3	-1.9	0.00187	0.196	0.078	12.1	0.3	12.2	0.4	[11]
14	33	0.3	1.1	4.6	11.5	0.7	0.02527	0.089	0.115	10.6	1.1	10.8	1.2	[12, 13]
15	7	1.9	2.1	3.1	8.2	-0.9	0.02270	0.149	0.232	8.7	0.61	9.1	0.86	[14]
16	41	0.2	2.3	3.6	18.4	0.5	0.01142	0.123	0.151	18	1	17.9	1.09	[15, 16]
17	30	1.1	3.4	2.8	7.6	-0.4	0.00640	0.185	0.156	7.6	0.7	7.9	0.7	[2]
18	33	1.7	3.7	1.5	9.1	-1.2	0.00929	0.159	0.192	10.2	0.68	10.3	0.87	[17]
19	41	1.6	4.2	2.6	9.5	-0.9	0.01075	0.163	0.196	10.2	0.87	10.4	0.64	[17]
20	31	0.7	5.7	2.5	12.2	-0.7	0.00447	0.105	0.067	13.1	1.48	12.9	1.27	[18]
21	11	0.4	1.3	3.0	8.2	-0.2	0.01756	0.052	0.225	8.3	0.37	8.4	0.6	[19]
22	11	1.1	1.4	3.9	14.0	-0.1	0.03560	0.107	0.408	14.1	0.8	NA	NA	[20]
23	33	1.2	1.9	2.5	13.7	0.5	0.00523	0.081	0.092	NA	NA	13.2	1.8	[21]
24	14	0.3	3.1	2.5	12.0	-1.1	0.00478	0.120	0.090	11.7	1.2	13.1	1.1	[11, 22]
25	18	0.4	1.8	2.1	9.3	-0.2	0.01712	0.095	0.267	9.7	0.49	9.5	0.65	[23]
26	18	2.2	3.1	2.3	9.3	-0.8	0.00818	0.152	0.173	9.8	0.81	10.1	0.86	[24]
27	63	1.0	2.4	2.6	11.3	-1.0	0.00534	0.125	0.066	11.8	1.75	12.3	1.76	[25]
28	31	0.7	1.2	3.0	11.8	0.6	0.01872	0.187	0.172	10.9	0.42	11.2	0.78	[26]
29	33	0.9	1.2	2.2	12.0	-0.3	0.04644	0.178	0.200	NA	NA	12.2	0.64	[11]
30	42	0.3	2.6	1.9	10.0	-0.2	0.00574	0.120	0.146	9.9	0.86	10.2	0.81	[27]
31	21	0.3	3.8	1.6	15.2	0.4	0.00218	0.175	0.142	14.8	0.7	NA	NA	[20]
32	61	0.8	1.4	2.0	12.7	-0.6	0.02195	0.160	0.230	13.3	0.6	13.3	0.6	[28, 29]
33	28	0.4	1.5	2.2	10.1	-1.1	0.02938	0.185	0.179	NA	NA	11.2	0.49	[30]
34	19	1.6	5.1	2.1	8.1	-0.6	0.00274	0.198	0.088	8.4	0.4	8.7	0.6	[2]
35	16	0.3	3.2	3.3	11.3	-1.6	0.00092	0.136	0.050	11.2	1.5	12.9	1.5	[5]
36	15	1.2	2.7	2.7	11.7	-0.4	0.01730	0.101	0.245	12.1	0.8	NA	NA	[20]
37	40	2.7	3.4	0.8	9.1	-0.6	0.00389	0.242	0.147	9.6	0.49	9.7	0.6	[31, 32]
38	25	1.2	4.4	2.3	10.3	-0.2	0.00259	0.158	0.098	10.1	0.97	10.5	1.1	[33]
39	22	1.1	4.5	2.8	7.0	-0.7	0.00430	0.131	0.115	7.7	0.9	NA	NA	[34]

References

1. Ogrinc N, Budja M (2005) Paleodietary reconstruction of a Neolithic population in Slovenia: a stable isotope approach. Chem Geol 218: 103–116.
2. Schurr MR, Powell ML (2005) The role of changing childhood diets in the prehistoric evolution of food production: an isotopic assessment. Am J Phys Anthropol 126: 278–294.
3. Pearson JA, Hedges REM, Molleson TI, Özbek M (2010) Exploring the relationship between weaning and infant mortality: an isotope case study from AşikliHöyük and Çayönü Tepesi. Am J Phys Anthropol 143: 448–457.
4. Weber AW, Link DW, Katzenberg MA (2002) Hunter-gatherer culture change and continuity in the Middle Holocene of the Cis-Baikal, Siberia. J Anthropol Archaeol 21: 230–299.
5. Howcroft R, Eriksson G, Lidén K (2012) Conformity in diversity? Isotopic investigations of infant feeding practices in two Iron Age populations from southern Öland, Sweden. Am J Phys Anthropol 149: 217–230.
6. Richards MP, Pearson JA, Molleson TI, Russell N, Martin L (2003) Stable Isotope Evidence of Diet at Neolithic Çatalhöyük, Turkey. J Archaeol Sci 30: 67–76.
7. Finucane B, Agurto PM, Isbell WH (2006) Human and animal diet at Conchopata, Peru: stable isotope evidence for maize agriculture and animal management practices during the Middle Horizon. J Archaeol Sci 33: 1766–1776.
8. Redfern RC, Hamlin C, Athfield NB (2010) Temporal changes in diet: a stable isotope analysis of late Iron Age and Roman Dorset, Britain. J Archaeol Sci 37: 1149–1160.
9. Redfern RC, Millard AR, Hamlin C (2012) A regional investigation of subadult dietary patterns and health in late Iron Age and Roman Dorset, England. J Archaeol Sci 39: 1249–1259.
10. Kusaka S, Ikarashi T, Hyodo F, Fujisawa S, Katayama K (2011) Stable isotope analysis on human skeletal remains from Edo-period Fushimi castle site in Japan. Anthropol Sci (Japanese Series) 119: 9–17.
11. Katzenberg MA, Saunders SR, Fitzgerald WR (1993) Age differences in stable carbon and nitrogen isotope ratios in a population of prehistoric maize horticulturists. Am J Phys Anthropol 90: 267–281.
12. Prowse T, Schwarcz HP, Saunders S, Macchiarelli R, Bondioli L (2004) Isotopic paleodiet studies of skeletons from the Imperial Roman-age cemetery of Isola Sacra, Rome, Italy. J Archaeol Sci 31: 259–272.
13. Prowse TL, Saunders SR, Schwarcz HP, Garnsey P, Macchiarelli R, et al. (2008) Isotopic and dental evidence for infant and young child feeding practices in an Imperial Roman skeletal sample. Am J Phys Anthropol 137: 294–308.
14. Bourbou C, Richards MP (2007) The Middle Byzantine menu: palaeodietary information from isotopic analysis of humans and fauna from Kastella, Crete. Int J Osteoarchaeol 17: 63–72.
15. Dupras TL (1999) Dining in the Dakhleh Oasis, Egypt: determination of diet using documents and stable isotope analysis. Ph.D. thesis, McMaster University.
16. Dupras TL, Schwarcz HP, Fairgrieve SI (2001) Infant feeding and weaning practices in Roman Egypt. Am J Phys Anthropol 115: 204–212.

17. Turner BL, Edwards JL, Quinn EA, Kingston JD, Van Gerven DP (2007) Age-related variation in isotopic indicators of diet at medieval Kulubnarti, Sudanese Nubia. Int J Osteoarchaeol 17: 1–25.

18. Keenleyside A, Schwarcz H, Stirling L, Lazreg NB (2009) Stable isotopic evidence for diet in a Roman and Late Roman population from Leptiminus, Tunisia. J Archaeol Sci 36: 51–63.

19. Triantaphyllou S, Richards MP, Zerner C, Voutsaki S (2008) Isotopic dietary reconstruction of humans from Middle Bronze Age Lerna, Argolid, Greece. J Archaeol Sci 35: 3028–3034.

20. Waters-Rist AL, Bazaliiskii VI, Weber AW, Katzenberg MA (2011) Infant and child diet in Neolithic hunter-fisher-gatherers from Cis-Baikal, Siberia: intra-long bone stable nitrogen and carbon isotope ratios. Am J Phys Anthropol 146: 225–241.

21. Clayton F, Sealy J, Pfeiffer S (2006) Weaning age among foragers at Matjes river rock shelter, South Africa, from stable nitrogen and carbon isotope analyses. Am J Phys Anthropol 129: 311–317.

22. Katzenberg MA (1993) Age differences and population variation in stable isotope values from Ontario, Canada. In: Lambert JB, Grupe G, editors, Prehistoric human bone: archaeology at the molecular level, New York: Springer Verlag. pp. 39–62.

23. Bocherens H, Polet C, Toussaint M (2007) Palaeodiet of Mesolithic and Neolithic populations of Meuse Basin (Belgium): evidence from stable isotopes. J Archaeol Sci 34: 10–27.

24. Williams JS, White CD, Longstaffe FJ (2005) Trophic level and macronutrient shift effects associated with the weaning process in the Postclassic Maya. Am J Phys Anthropol 128: 781–790.

25. Richards MP, Fuller BT, Molleson TI (2006) Stable isotope palaeodietary study of humans and fauna from the multi-period (Iron Age, Viking and Late Medieval) site of Newark Bay, Orkney. J Archaeol Sci 33: 122–131.

26. Choy K, Richards MP (2009) Stable isotope evidence of human diet at the Nukdo shell midden site, South Korea. J Archaeol Sci 36: 1312–1318.

27. Fuller BT, Molleson TI, Harris DA, Gilmour LT, Hedges REM (2006) Isotopic evidence for breastfeeding and possible adult dietary differences from Late/Sub-Roman Britain. Am J Phys Anthropol 129: 45–54.

28. Nitsch EK, Humphrey LT, Hedges REM (2010) The effect of parity status on δ^{15}N: looking for the “pregnancy effect” in 18th and 19th century London. J Archaeol Sci 37: 3191–3199.

29. Nitsch EK, Humphrey LT, Hedges REM (2011) Using stable isotope analysis to examine the effect of economic change on breastfeeding practices in Spitalfields, London, UK. Am J Phys Anthropol 146: 619–628.

30. Tuross N, Fogel ML (1994) Stable isotope analysis and subsistence patterns at the sully site. In: Owsley D, Jantz R, editors, Skeletal biology in the Great Plains: migration, warfare, health, and subsistence, Washington: Smithsonian Institution Press. pp. 283–289.

31. Jay M, Richards MP (2006) Diet in the Iron Age cemetery population at Wetwang Slack, East Yorkshire, UK: carbon and nitrogen stable isotope evidence. J Archaeol Sci 33: 653–662.

32. Jay M, Fuller BT, Richards MP, Knüsel CJ, King SS (2008) Iron Age breastfeeding practices in Britain: isotopic evidence from Wetwang Slack, East Yorkshire. Am J Phys Anthropol 136: 327–337.

33. Choy K, Jeon OR, Fuller BT, Richards MP (2010) Isotopic evidence of dietary variations and weaning practices in the Gaya cemetery at Yeanri, Gimhae, South Korea. Am J Phys Anthropol 142: 74–84.

34. Gardner KS, Leventhal A, Cambra R, Bartelink EJ, Martinez A (2011) Mothers and infants in the prehistoric Santa Clara Valley: what stable isotopes tell us about ancestral Ohlone weaning practices. SCA Proceedings 25: 1–14.
