# Supplementary material for: Quantitative Reconstruction of Weaning Ages in Archaeological Human Populations Using Bone Collagen Nitrogen Isotope Ratios and Approximate Bayesian Computation
Source: PLoS One. 2013 Aug 27;8(8):e72327. doi: 10.1371/journal.pone.0072327 (PMC3754991; doi:10.1371/journal.pone.0072327)
Supplement: Text S1 — Detailed procedures and mathematical expressions in the estimation of subadult bone collagen turnover rate and the WARN program. (PDF) [file pone.0072327.s003.pdf]

Text S1. Detailed procedures and mathematical expressions in the estimation of subadult bone collagen turnover rate and the WARN program.

1 Bone collagen turnover rate

Basic functions to describe the temporal changes of bone mineral were derived from several previous studies. Following Mitchell et al. [1], the bone mineral mass $C(t)$ at age of t years was represented as:
$C(t) = 28.0 + 86.828t - 16.5105t^2 + 1.5625t^3 - 0.04114t^4$ $(0 \geq t \geq 20)$ (Equation S1-1).
This equation represents the modeling process of bone turnover. On the other hand, the remodeling rate $\gamma(t)$ at age of t years was represented as follows:

1. $\gamma(t) = \frac{104.3}{C(t)}$, when $t \leq 1.5$, and

2. $\gamma(t) = 0.975\mathrm{e}^{-0.11t}$, when $t > 1.5$ (Equation S1-2).

This equation was obtained from Leggett et al. [2] and was derived from direct histological observations of subadult rib bone formation and resorption performed by Frost [3]. Note that a term for the radioactive decay of ^{90}Sr (0.025 per year), included in the original equations, was excluded from our equations. Following Ruffoni et al. [4], the mineralization law $\lambda(i)$, which describes the rate of mineralized collagen portion at ith years after the collagen matrix was formed, was set as:
$\lambda(i) = c_1 \frac{1+\frac{i}{i_1}}{\frac{i}{i_1}} + c_2 \frac{1+\frac{i}{i_2}}{\frac{i}{i_2}}$ (Equation S1-3).
In this study, c_1, i_1, c_2 and i_2 were set as 18/23, 1/300, 25/92, and 5, respectively. Equation S1-3 corresponds to over 70% primary mineralization in a few days and protracted secondary mineralization of up to 100% in about 20 years, values that have been given in several previous studies [4–7].

Temporal changes in the bone mineral turnover rate can be represented using these functions. Put simply, the bone mineral turnover rate, $T_{min}[t]$, over one unit of time from $t-1$ to t years, was represented as follows:
$T_{min}[t] = \frac{C(t)-C(t-1)}{C(t)} + \int_{t-1}^{t} \gamma(x)dx \frac{C(t-1)}{C(t)}$ (Equation S1-4).
The former and latter terms in the function indicate the effects of bone modeling and remodeling, respectively. Using the bone collagen turnover rate, $T_{col}[t]$, over one unit of time from $t-1$ to t years, $T_{min}[t]$ can also be represented as follows:

1. $T_{min}[t] = T_{col}[t]\Delta\lambda[1]$, when $t = 1$, and

2. $T_{min}[t] = T_{col}[t]\Delta\lambda[1] + \sum_{j=1}^{t-1}(T_{col}[j]\frac{C(j)}{C(t)}\Delta\lambda[t+1-j])$, when $t \geq 2$ (Equation S1-5).

The former and latter terms in the second function shown in Equation S1-5 indicate the effects of turnover delay in the bone mineral for the intended unit of time (i.e., $t-1$ to t years) and the aggregated effects of the delay for the former unit times (i.e., 0 to 1 year, 1 to 2 years, .., and $t-2$ to $t-1$ years). The turnover rate over one unit of time (i.e., one year) from $t-1$ to t years can be sequentially calculated using Equation S1-4 and S1-5, as indicated in Table 2. The resulting discrete turnover rates were coerced to a quartic polynomial formula using the *nls* function in R, in accordance with the quartic formula for bone mineral mass (i.e., Equation S1-1). The formula is represented as follows:
$T_{col}[t] = 1.778 - 0.4121t + 0.05029t^2 - 0.002756t^3 + 0.0005325t^4$ (Equation S1-6),
and is shown in Figure 2.

2 Changes in diet and bone collagen δ^{15}N values

Following Millard [8], the δ^{15}N values for newly synthesized collagen $\delta^{15}N_{new}(t)$ at a given age of t years are given by the following equation:
$\delta^{15}N_{new}(t) = (1 - p(t))(\delta^{15}N_{mother} + E) + p(t)\delta^{15}N_{wnfood}$ (Equation S2-1).
The proportion of non-milk protein in the total dietary protein intake at age of t is represented as $p(t)$. The δ^{15}N value for the mother's milk is described as $\delta^{15}N_{mother} + E$, using the δ^{15}N value for the mothers tissue, $\delta^{15}N_{mother}$ (approximated by the mean δ^{15}N value for adult females), and a ^{15}N enrichment factor for the transfer from the maternal to infant tissue, E. The δ^{15}N value for collagen synthesized from non-milk foods is represented as $\delta^{15}N_{wnfood}$. We considered $\delta^{15}N_{wnfood}$ to be a variable because children in the past could have eaten supplementary foods with different δ^{15}N values from the adult mean δ^{15}N values. This value has been approximated in previous studies as the mean δ^{15}N value for the adults.

The proportion of non-milk protein in the total dietary protein intake is assumed, in our model, to increase exponentially. The relative proportion of non-milk protein at the age of t years, $p(t)$, is described as follows:

1. $p(t) = 0$, when $t < t_1$ (breast milk only),
2. $p(t) = (\frac{t-t_1}{t_2-t_1})^2$, when $t_1 \leq t \leq t_2$ (during the weaning process), and
3. $p(t) = 1$, when $t > t_2$ (no breast milk), (Equation S2-2),

where the ages at the start and end of weaning are represented as t_1 and t_2, respectively. Equation S2-2 was derived from a model proposed by Millard [8] and represents slow initial weaning and rapid final weaning. In the original model, four forms (linear, parabolic, reverse parabolic, and sigmoid) of dietary change were applied to condition 2 in Equation S2-2. Although the form of dietary change during

weaning can be selected in the WARN package, we used only the parabolic form because Millard [8] used a parabolic weaning pattern to model the changes in δ^{15}N in archaeological datasets. This seems to be a reasonable assumption as not only the amount of milk protein consumed decreases during the weaning process but also the proportion of milk protein consumed also decreases because of the increasing total dietary intake in growing subadults.

The δ^{15}N value for bone collagen at the age of t years, $\delta^{15}N_{bone}(t)$, is calculated as follows:
$\delta^{15}N_{bone}(t) = \delta^{15}N_{bone}(t-1)(1 - T_{col}[t]) + \int_{t-1}^{1} \delta^{15}N_{new}(t)(x)dxT_{col}[t]$ (Equation S2-3).
The former and latter parts of the equation represent the remaining and the newly synthesized portion, respectively, of the bone collagen over one unit of time from $t-1$ to t years. Extending equation S2-3, the δ^{15}N value for bone collagen at the age of $t+a$, i.e., a being a part of one year from the unit time point t $(0 < a < 1)$, is represented as:
$\delta^{15}N_{bone}(t+a) = \delta^{15}N_{bone}(t)(1 - T_{col}[t+a]) + \int_{t}^{t+a} \delta^{15}N_{new}(t)(x)dxT_{col}[t+a]$ (Equation S2-4).
In equation S2-4, $T_{col}[T]$ is the bone collagen turnover rate over a year from t to $t+a$, given by:
$T_{col}[t+a] = T_{col}[t+1]\frac{\int_{t}^{t+a} T_{col}[x]dx}{\int_{t}^{t+1} T_{col}[x]dx}$ (Equation S2-5).
The bone collagen δ^{15}N values for each unit of time (one year) can be calculated sequentially, as reference values, using Equation S2-3 under the given parameters. The δ^{15}N values that correspond to the observed ages for the samples can then be calculated from the reference values and Equation S2-4. The initial bone collagen values at 0 year of age, $\delta^{15}N_{bone}(0)$, were approximated using the mean δ^{15}N value of adult females, because the δ^{15}N value for infant tissue is assumed to be the same as to that of the mother [9]. Theoretical δ^{15}N values for the age of each individual in the observed dataset can be calculated using Equation S2-4.

In our model, the differences between the individuals are evaluated by calculating mean square distance, D, between the observed and simulated δ^{15}N values. Put simply, point estimates of the parameters with minimized D can be calculated by solving the optimization problem (the application of the optimization problem to palaeo dietary reconstructions has been described by Little and Little [10]). These represent point estimates under the framework of maximum likelihood estimates (MLE). Although the point estimates do not provide information on the error ranges, they will be used later in the SMC sampling procedures; therefore, optimized values for weaning parameters under the MLE framework were calculated. We used the *optim* function in R to obtain the optimized parameter value, θ_{opt}, and its resultant minimum mean square distance, D_{opt}.

3 SMC sampling procedures

SMC sampling is characterized by a successive reduction in tolerance and a weighted resampling from the previous parameter population, called a "particle". Particles of preliminary simulations are used to calculate the next set of parameter vectors, to generate simulated data within a certain distance D from the observed data. The particles are then repeatedly resampled (according to a weighting scheme that considers the prior distributions), perturbed (using a transition kernel), and judged (on the basis of a successively decreasing tolerance). The particles after this iterative process finally approximate a sample of the posterior distribution of the parameters. In particular, the partial rejection control procedures prune away parameters that have minimal impacts on the final estimation in the parameter weighting step in the earlier stages of the tolerance reduction, and this increases the sampling efficiency [11].

To adopt the ABC framework, we added individual error terms ϵ_i in Equation S2-4 as follows: $\delta^{15}N_{bone}(t+a) = \delta^{15}N_{bone}(t)(1 - T_{col}[t+a]) + \int_t^{t+a} \delta^{15}N_{new}(x)dxT_{col}[t+a] + \epsilon_i$ (Equation S3-1). These errors were independently sampled from the normal distribution with mean of 0.0 and SD of σ, and individually assigned to simulated δ^{15}N values. By considering this individual error term, parameters that result in D values smaller than D_{opt} can be generated, which represent more plausible estimates for the measured data. In the ABC framework, D values are calculated using randomly generated parameters from the prior distributions, then the parameters that result in D values smaller than D_{opt} become the posterior distributions.

The sequential Monte Carlo algorithm in our model proceeds as follows (see Sisson et al. [12] for more details):

1. Set prior distributions $\pi(\cdot)$ for the parameters and the number of particles j in one population. Calculate the final tolerance α_K $(= D_{opt})$ under the MLE framework and set decreasing tolerances. Set the population indicator $k = 1$ (initialization).

2. Set the particle indicator $j = 1$ (initialization).

   (a) If $k = 1$, independently sample θ^{**} from the prior distribution $\pi(\theta)$. If $k > 1$, sample θ^{*} from the previous population $\theta_{k-1}^{(i)}$ with weights $W_{k-1}^{(i)}$, and perturb the particle to θ^{**} with transition kernel ϕ. Simulate the change in the δ^{15}N value $\delta^{15}N_{new}^{**}(t)$ with θ^{**} using equation S3-1. If $D^{**} - D_{opt} \geq \alpha_k$, θ^{**} are rejected and then repeat procedure 2(a).

   (b) Set the indicators as follows:

   - $\theta_k^{(j)} = \theta^{**}$,
   - $W_k^{(j)} = 1$ (if $k = 1$), and

- $W_k^{(j)} = \frac{\pi(\theta_{k-1}^{(j)})}{\sum_{x=1}^{J} W_{k-1}(\theta_{k-1}^{(x)})\phi(\theta_k^{(j)}|\theta_{k-1}^{(x)})}$ (if $k > 1$).

  If $j < J$, increment $j = j + 1$ and go to procedure 2(a).

3. Normalize the weights so that:
   $\sum_{j=1}^{J} W_k^{(j)} = 1$.
   If the requirements for an effective sample size ESS are not met such as:
   $ESS = \frac{1}{\sum_{j=J}^{J} W_k^{(j)2}} < \frac{I}{2}$,
   sample with replacement, the particles $\theta_k^{(j)}$ with weights $W_k^{(j)}$ to obtain a new population $\theta_k^{(j)}$, and set weights $W_k^{(j)} = \frac{1}{J}$.

4. If $k < K$, increment $k = k + 1$ and go to procedure 2.

Prior distributions $\pi(\cdot)$ were set as normal distributions with default means of $\{0.5, 3.0, 1.9, \delta^{15}N_{mother}$, and 0.0$\}$ and SDs of $\{3.0, 3.0, 0.9, 3.0$, and 1.0$\}$ for t_1, t_2, E, $\delta^{15}N_{wnfood}$, and σ, respectively. The mean weaning age was obtained from values recommended by modern pediatricians and the biologically expected ages [13]. The mean and standard deviation of the enrichment factor E was obtained from the values reported by Waters-Rist and Katzenberg [14]. The hyper parameter for the individual error term σ was used as an absolute value in the calculation. The default number of particles J was 10000. Decreasing tolerances α_k were set as $D_{opt} + \{2, 1, 0.5, 0.25, 0.125, 0.0625, 0\}$ and, therfore, the number of populations $K = 7$. The transition kernel ϕ was set to be a normal distribution with a mean of 0.0 and SD of 0.1.

References

1. Mitchell HH, Hamilton TS, Steggerda FR, Bean HW (1945) The chemical composition of the adult human body and its bearing on the boichemistry of growth. J Biol Chem 158: 625–637.

2. Leggett RW, Eckerman KF, Williams LR (1982) Strontium-90 in bone: a case study in age-dependent dosimetric modeling. Health Physics 43: 307–322.

3. Frost HM (1969) Tetracycline-based histological analysis of bone remodeling. Calcif Tissue Res 3: 211–237.

4. Ruffoni D, Fratzl P, Roschger P, Klaushofer K, Weinkamer R (2007) The bone mineralization density distribution as a fingerprint of the mineralization process. Bone 40: 1308–1319.

5. Akkus O, Polyakova-Akkus A, Adar F, Schaffler MB (2003) Aging of microstructural compartments in human compact bone. J Bone Miner Res 18: 1012–1019.

6. Fratzl P, Gupta HS, Paschalis EP, Roschger P (2004) Structure and mechanical quality of the collagen-mineral nano-composite in bone. J Mater Chem 14: 2115–2123.

7. Ruffoni D, Fratzl P, Roschger P, Phipps R, Klaushofer K, et al. (2008) Effect of temporal changes in bone turnover on the bone mineralization density distribution: a computer simulation study. J Bone Miner Res 23: 1905–1914.

8. Millard AR (2000) A model for the effect of weaning on nitrogen isotope ratios in humans. In: Goodfriend GA, Collins MJ, Macko SA, Wehmiller JF, editors, Perspectives in amino acid and protein geochemistry, New York: Oxford University Press. pp. 51–59.

9. Fuller BT, Fuller JL, Harris DA, Hedges REM (2006) Detection of breastfeeding and weaning in modern human infants with carbon and nitrogen stable isotope ratios. Am J Phys Anthropol 129: 279–293.

10. Little JDC, Little EA (1997) Analysing prehistoric diets by linear programming. J Archaeol Sci 24: 741–747.

11. Liu JS (2001) Monte Carlo strategies in scientific computing. New York: Springer Verlag.

12. Sisson SA, Fan Y, Tanaka MM (2007) Sequential Monte Carlo without likelihoods. Proc Nat Acad Sci 104: 1760–1765.

13. Dettwyler KA (2004) When to wean: biological versus cultural perspectives. Clin Obstet Gynecol 47: 712–723.

14. Waters-Rist AL, Katzenberg MA (2010) The effect of growth on stable nitrogen isotope ratios in subadult bone collagen. Int J Osteoarchaeol 20: 172–191.
